# Longitudinal Study of Two Irish Dairy Herds: Low Numbers of Shiga Toxin-Producing *Escherichia coli* O157 and O26 Super-Shedders Identified

**DOI:** 10.3389/fmicb.2016.01850

**Published:** 2016-11-18

**Authors:** Brenda P. Murphy, Evonne McCabe, Mary Murphy, James F. Buckley, Dan Crowley, Séamus Fanning, Geraldine Duffy

**Affiliations:** ^1^Veterinary Department, Veterinary Food Safety Laboratory, Cork County Council, County CorkIreland; ^2^Teagasc Food Research Centre, AshtownIreland; ^3^UCD Centre for Food Safety, School of Public Health, Physiotherapy and Sports Science, University College DublinDublin, Ireland

**Keywords:** STEC, super-shedding, raw milk, cattle, recto-anal, swabs

## Abstract

A 12-month longitudinal study was undertaken on two dairy herds to ascertain the Shiga-toxin producing *Escherichia coli* (STEC) O157 and O26 shedding status of the animals and its impact (if any) on raw milk. Cattle are a recognized reservoir for these organisms with associated public health and environmental implications. Animals shedding *E. coli* O157 at >10,000 CFU/g of feces have been deemed super-shedders. There is a gap in the knowledge regarding super-shedding of other STEC serogroups. A cohort of 40 lactating cows from herds previously identified as positive for STEC in a national surveillance project were sampled every second month between August, 2013 and July, 2014. Metadata on any potential super-shedders was documented including, e.g., age of the animal, number of lactations and days in lactation, nutritional condition, somatic cell count and content of protein in milk to assess if any were associated with risk factors for super-shedding. Recto-anal mucosal swabs (RAMS), raw milk, milk filters, and water samples were procured for each herd. The swabs were examined for *E. coli* O157 and O26 using a quantitative real time PCR method. Counts (CFU swab^-1^) were obtained from a standard calibration curve that related real-time PCR cycle threshold (*C*_t_) values against the initial concentration of O157 or O26 in the samples. Results from Farm A: 305 animals were analyzed; 15 *E. coli* O157 (5%) were recovered, 13 were denoted STEC encoding either *stx1* and/or *stx2* virulence genes and 5 (2%) STEC O26 were recovered. One super-shedder was identified shedding STEC O26 (*stx1*&2). Farm B: 224 animals were analyzed; eight *E. coli* O157 (3.5%) were recovered (seven were STEC) and 9 (4%) STEC O26 were recovered. Three super-shedders were identified, one was shedding STEC O157 (*stx2*) and two STEC O26 (*stx2*). Three encoded the adhering and effacement gene (*eae)* and one isolate additionally encoded the haemolysin gene (*hlyA*). All four super-shedders were only super-shedding once during the 1-year sampling period. The results of this study show, low numbers of super-shedders in the herds examined, with high numbers of low and medium shedding. Although four super-shedding animals were identified, no STEC O157 or O26 were recovered from any of the raw milk, milk filter, or water samples. The authors conclude that this study highlights the need for further surveillance to assess the potential for environmental contamination and food chain security.

## Introduction

Shiga-toxin producing *Escherichia coli* (STEC) O157 and O26 are well known human pathogens; emerging in the last 20 years as a major cause of illness and a serious public health issue (O’Brien et al., 1983; MacDonald et al., 1988). There were 704 cases of STEC in Ireland in 2014 of those 178 were caused by STEC O157 and 233 by STEC O26 (Health Protection Service Centre [HPSC], 2014). The European Food Safety Authority annual zoonoses reports show that, Ireland had the highest numbers of human cases of STEC in the EU in 2012–2014 (8.99, 12.29, and 12.42/100,000 population, respectively) (European Food Safety Authority [EFSA], 2014, 2015, 2016). The relevance of these pathogens is related to their low infective dose (10 – 100 bacteria) and the severity of the disease they cause (Karch et al., 2005). Foodborne contamination levels as low as 3–15 viable cells per gram of beef and 3–4 viable cells per 10 g of Salami have been associated with major outbreaks (Rangel et al., 2005). STEC infections in humans can cause three severe syndromes, hemorrhagic colitis (HC), haemolytic-uremic syndrome (HUS), and/or thrombotic thrombocytopenic purpura (TTP) (Mainil and Daube, 2005). Although STEC O157 was shown in a recent study on Irish cattle to be the predominant serotype shed in with bovine feces (2.3%), a range of other STEC serogroups including. O26 (1.5%), O103 (1.0%), O145 (0.7%) were also recovered (Thomas et al., 2012; European Food Safety Authority [EFSA], 2014).

The main virulence characteristic of STEC strains is the production of Shiga-toxins (denoted *stx* 1 and 2) (Sandvig, 2001). It has been reported that *stx2* and its variants are more likely to cause severe human disease (Caprioli et al., 2005; Persson et al., 2007; Bondari et al., 2015). Additionally, some isolates associated with human disease possess an adhering and effacement *(eae*) gene, responsible for the histopathological lesion, located on the pathogenicity island known as the locus of enterocyte effacement (LEE). Some strains may also possess a further virulence determinant haemolysin (*hlyA*) a plasmid-encoded enterohemolysin (Bondari et al., 2015).

It is generally accepted that cattle and other ruminant animals are a major reservoir of transmission with many infections originating either indirectly or directly from exposure to cattle feces (Karmali, 2004). Cattle are asymptomatic carriers of STEC as they lack the Gb3 receptor on their cell surface and the toxins cannot bind (Pruimboon-Brees et al., 2000; Bondari et al., 2015), thus, presenting no clinical symptoms while shedding these bacteria. Intermittent or persistent fecal shedding may occur following repeated exposure to the organism, through contaminated environmental sources or ingestion of contaminated water and feedstuffs (Rice et al., 2003; Carlson et al., 2009).

A small proportion of *E. coli* O157 positive animals shed the organisms at higher levels than others, these animals are denoted ‘super-shedders’ (shedding ≥10,000 CFU/g of feces) (Matthews et al., 2006a; Chase-Topping et al., 2007, 2008; Cobbold et al., 2007; Stanford et al., 2012; Arthur et al., 2013; Munns et al., 2015). Menrath et al. (2010) studied the phenomenon of super-shedding non-O157 serotypes and reported some significant risk factors for shedding STEC; the month of sampling (prevalence is higher in August, September, and October); the number of lactations (first-time calvers) and days in milk (50 – 150 days or more than 350 days); the nutritional condition (higher than 3.5); the somatic cell count (lower than 100,000 cells/ml) and the content of protein in milk (higher than 3.0%).

As STEC may be part of the microbiota of the healthy animal, raw milk contamination may occur, inadvertently, during the milking process. Illness associated with the consumption of raw milk is rare in Ireland, as almost all liquid milk consumed is now pasteurized. However, there is documented evidence globally of the risks associated with raw milk consumption due to the possibility of STEC (and other pathogen) contamination in raw milk and raw milk products (Allerberger et al., 2003; Murphy et al., 2005, 2007; Rangel et al., 2005; Schrijver et al., 2008; Lynch et al., 2011; Madic et al., 2011; Pennington, 2014; FSAI Report, 2015). Diverse opportunities for contamination of raw milk exist, e.g., animal to animal contact and environmental sources (fomites, vectors, aerosols) (Ferens and Hovde, 2011). Humans and equipment present on the farm also pose a risk. To maintain the integrity of the production unit, necessary sanitation rules must be enforced this includes the use of disinfectants at key points and the wearing of protective clothing and footwear. Standard bio-security practices including rodent and pest controls together with effective controls on the hygienic quality of feedstuffs and water sources will help to reduce the potential health risk at farm level (Collins and Wall, 2004). However, the presence of super-shedders in a herd may have a disproportionately high impact on the risk of transmission on the farm, in the food chain and for the environment (water catchment areas in particular) (Rangel et al., 2005; Chase-Topping et al., 2008).

Several studies have confirmed that the principal colonization site for these bacteria in cattle is the recto-anal junction (RAJ) (Naylor et al., 2003; Rice et al., 2003; Davis et al., 2006; Cobbold et al., 2007; Nart et al., 2008; Carlson et al., 2009; Arthur et al., 2013). RAMS are deemed to be a sensitive method for the detection of STEC in cattle (Rice et al., 2003; Davis et al., 2006). In this study, RAMS were used as the sample matrix for quantitation, to determine the shedding status of individual animals. Raw milk, milk filter, and water samples were also screened for the pathogens.

Cattle super-shedding STEC increase the risk of transmission of this pathogen in the farm environment and into dairy (unpasteurized milk and farm house cheese) and the beef chain. There is a gap in the knowledge on the frequency of super-shedding and the factors causing it. Identifying such animals will lead to control measures for example; segregation from the food chain or introducing targeted interventions. This study provided preliminary information on super-shedding in Irish Dairy herds, thereby feeding into guidelines on the management of such super-shedding animals. It is hypothesized that the phenomenon of super-shedding may be related to intermittent modulations in the resident micro-flora of the RAJ, allowing *E. coli* O157 (or O26) to flourish and dominate in some animals for a period of time or maybe as a consequence of genetic variations in different STEC strains (Cobbold et al., 2007: Jeon et al., 2013; Cote et al., 2015). We hypothesize that the presence of STEC super-shedders is directly related to the presence of these pathogens in the milk tank.

The objective of this study was to investigate the presence of active super-shedders in Irish herds and its impact (if any) on raw milk contamination.

## Materials and Methods

### Herd Selection

This study was undertaken on two dairy herds in Ireland over 12 months. The herds were selected out of 18 positive herds that had participated in a National Surveillance Project, investigating the prevalence of STEC O157 and O26 in raw milk and milk filters (FSAI Report, 2015). The design of this study was longitudinal with the intent to determine the shedding status of particular animals in a herd over time, including low-shedders (1–10 CFU/swab), medium-shedders (100 – 1000 CFU/swab), and particularly the presence (if any) of super-shedders (cows shedding ≥10,000 CFU/swab) and the impact of this phenomenon on potential raw milk contamination.

### Animal Selection

The number of animals to be screened was determined using criteria set out by Cannon and Roe (1982). Forty lactating animals from each herd, to be sampled were chosen. The same 40 animals were sampled at each visit to the farm, where possible (herd population size 70; 5% expected proportion of super-shedders in the population; 95% confidence of identifying at least one super-shedder) (Cannon and Roe, 1982). Metadata collected from animals examined for the presence of Shiga-toxin producing-producing *E. coli* in bovine feces were included (Menrath et al., 2010).

### Sample Collection

The dairy herd owners participated voluntarily and were assured of confidentiality. At the outset of the study a questionnaire was completed by each herd owner to gather information regarding herd size; animal husbandry; on-farm hygiene practices; feed type; water supply type; family age group and raw milk consumption practices on the premises. The recto-anal swabs from each individual animal (which was then designated a sample code) were procured by the herd’s private veterinary practitioner (PVP) with the assistance of a local authority veterinary surgeon during the milking process. The milk filter used during the milking session was taken for analysis. The raw milk sample was taken, aseptically, from the lower valve of the tank and the water sample was taken as per the European Union (Drinking Water) Regulations (2014) (Statutory Instrument No. 122/2014). The samples were returned to the laboratory within 4 h at 4°C in a temperature controlled container.

### Isolation and Characterisation of STEC O157 and O26

#### Enrichment and DNA Extraction

On return to the laboratory the samples were refrigerated overnight at 4°C, the following morning, the samples were vortexed for 2 min and incubated for 5 h in Tryptone Soya Broth (Oxoid, Basingstoke, Hampshire, England) with novobiocin (Oxoid) [20 μg/ml] (mTSB) at 41.5 ± 1°C. Post-incubation an aliquot of 1 ml was transferred to a 1.5 ml eppendorf for DNA extraction using DNeasy Blood and Tissue Kit, as per manufacturers’ instructions (Qiagen GmbH, Hilden, Germany).

#### Real-Time Polymerase Chain Reaction

Samples were examined for *E. coli* O157 and O26 using a quantitative real time PCR method following an initial enrichment (Lawal et al., 2015). Counts (CFU swab^-1^) were obtained from a standard calibration curve that related the real-time PCR cycle threshold (*C*_t_) values to the initial concentration of O157 or O26 in the samples. The calibration curve was set up and validated on a Rotor-Gene 6000 instrument using spiked naturally contaminated swabs previously found negative for the presence of STEC (*n* = 150) which were inoculated with EDL933 *E. coli* O157 and NFC361 *E. coli* O26 at 10^1^–10^7^ CFU/swab.

#### Culture Methods

In the event of a positive result from the real time PCR the samples was culturally examined to obtain an isolate. Immuno-magnetic separation (IMS) was performed using Dynabeads anti-*E. coli* O157 or O26 as per manufacturers’ instructions (*life* technologies, Oslo, Norway). After IMS the bead-bacteria complex was resuspended in 50 μl of phosphate buffered saline (PBS, Fannins-LIP, Galway, Ireland) and plated onto Cefixime-Tellurite Sorbitol MacConkey Agar (CT-SMAC) and ChromAgar O157 (Fannins-LIP) for *E. coli* O157 detection and in duplicate onto Cefixime-Tellurite Rhamnose MacConkey Agar (CT-RMAC, Lab M, Lancashire, UK) for *E. coli* O26 detection. Plates were incubated at 37 ± 1°C for 18 to 24 h. When present, up to five typical colonies from CT-SMAC and CT-RMAC were carried forward for confirmation tests. Typical colonies were subjected to a slide agglutination test conducted with single antisera (O157 and/or O26; Statens Serum Institut, Copenhagen, Denmark) and screened for the presence of indole production (ProLab Diagnostics, Bromborough, UK) (ISO 16654, 2001). All work was performed in a Category 3 facility within the Cork County Council campus, protocols and standard operating procedures were strictly adhered to for the duration of the study. All positive strains were stocked at both -20 and -80°C and are held at the Veterinary Food Safety Laboratory.

#### Virulence Determination

DNA extraction from the cultures was performed using a DNeasy Blood and Tissue Kit (Qiagen, Crawley, West Sussex, UK) as per manufacturers’ instructions. Nucleic acid amplification tests using real-time PCR were applied to the extracted DNA to determine the virulence status. The assay targets were the four common virulence genes of STEC O157 and O26 (*stx1*, *stx2*, *eae*, and *hlyA*). Two duplex real-time PCR’s were employed, one to amplify *stx1* and *eae* (ISO/TS 13136, 2012) and the other to amplify *stx2* and *hlyA* (This Study; Accession No: AB779751.1 and Accession No: AY278115.1, respectively). Sequence searches were carried out using the BLAST program available at the NCBI BLAST home page^1^ (Altschul et al., 1997). Probes, primers, associated fluorescent and quencher dyes are shown on **Table 1**. Additional reagents and final concentrations were as follows: *Taq* JumpStart mix [1X] (Sigma–Aldrich, St. Louis, MO, USA); MgCl_2_ [25 mM] (Sigma–Aldrich); Bovine Serum Albumin [50 ug/ml] (Thermo Fisher Inc., Waltham, MA, USA); Exogeneous Internal Positive Control (IPC) mix [10X] and IPC DNA were used as per manufacturers’ instructions (*life* technologies). All probes (*stx1*, *stx2*, *eae*, and *hlyA*) were at a concentration of 5 pM. The *stx1* primers were used at 20 pM and *eae* primers at 10 pM (ISO/TS 13136, 2012), the *stx2 and hlyA* primers at 10 pM (this study). Molecular grade DNAse and RNAse free water (Roche GmbH, Mannheim, Germany) was added to bring the volume to 24 and 1 μl of template DNA [10 ng/μl] to a final volume of 25 μl/tube. RT-PCR conditions were as follows: hold at 94°C for 2 min; cycling 95°C for 15 s; 60°C for 60 s repeat 30 times. Probes and primers developed for this study were designed using an online bioinformatic tool, GenScript Real-time PCR (*Taq*man) Probe/Primer Design^2^. All primers and probes were generated by Eurofins Genomics, Regensberg, Germany^3^.

**Table 1:** Probes and primers used for amplification of virulence genes in real-time PCR assays (ISO/TS 13136, 2012 and this study).

*Oligo name*	*5′ -Label*	*Primer sequence <5′ → 3′>*	*3′-Label*	*Reference*
*stx*1- Probe	FAM	CTG GAT GAT CTC AGT GGG CGT TCT TAT GTA A	BHQ1	ISO/TS 13136, 2012
*stx*1- Forward		TTT GTT ACT GTG ACA GCT GAA GCT TTA CG		
*stx*1- Reverse		CCC CAG TTC AAT GTA AGA TCA ACA TC		
*eae*- Probe	ROX	ATA GTC TCG CCA GTA TTC GCC ACC AAT ACC	BHQ2	
*eae*- Forward		CAT TGA TCA GGA TTT TTC TGG TGA TA		
*eae*- Reverse		CTC ATG CGG AAA TAG CCG TTA		
*stx*2- Probe	FAM	CTG TCT GAA ACT GCT CCT GTG	BHQ1	This study
*stx*2- Forward		CCA GTT CAG AGT GAG GTC CA		
*stx*2- Reverse		TCA GTT CGA TAC CCG CTG CAG C		
*hyl*A-Probe	ROX	TCT CCG GAA TTC TTT CTG CT	BHQ2	This study
*hyl*A-Forward		GCG AAA CAG CTT TAC CAA CA		
*hyl*A-Reverse		CGTC TCC CGG CGTC ATC GTA		

## Results

A total of 529 RAMS were analyzed for the presence of STEC O157 and O26 over a 12-month period. Farm A was visited eight times and Farm B was visited six times between August, 2013 and July, 2014 (**Tables 2** and **3**). Three hundred and five individual swabs were sampled from Farm A and 224 from Farm B.

**Table 2:** Farm A: Month of sampling, numbers of animals sampled, animal code, number of lactations, age of animal, *E. coli* serogroup isolated, shedding status, and virulence characteristics.

Month of sampling	No. Lactating animals sampled	Animal code	No. of lactations	Age of animal	*E. coli* serogroup isolated	Shedding status CFU/swab	Virulence status of recovered isolates			
							*stx*1	*stx*2	*eae*	*hly*A
August, 2013	38	VFSL434	3	4y7m	O157	10	–	+	+	–
November, 2013	37	VFSL364	5	6y7m	O26	10	–	+	+	–
		VFSL731^∗^	2	3y5m	O26	10	–	+	+	+
		VFSL432	3	4y7m	O157	<10	–	+	+	–
		VFSL531^∗^	2	3y1m	O157	<10	–	+	+	–
		VFSL331	6	7y7m	O157	<10	–	+	+	–
		VFSL435	3	4y7m	O157	<10	–	+	+	–
January, 2014	35	VFSL887^∗^	1	2y6m	O157	<10	–	+	+	–
		VFSL537	2	3y3m	O26	10,000	–	+	+	–
		VFSL473^∗^	4	6y8m	O157	<10	–	–	+	–
February, 2014	39	VFSL703	2	3y3m	O157	<10	+	+	+	–
		VFSL887^∗^	1	2y7m	O157	<10	–	+	+	–
March, 2014	40	VFSL165	11	12y7m	O157	<10	–	+	+	–
		VFSL434	3	5y2m	O157	<10	–	–	+	–
		VFSL887^∗^	1	2y8m	O157	10	–	+	+	–
April, 2014	40	No STEC Detected								
May, 2014	38	VFSL326	6	7y7m	O157	<10	–	+	+	–
		VFSL405	3	5y7m	O157	<10	–	+	+	–
		VFSL531^∗^	2	3y7m	O157	<10	–	+	+	–
July, 2014	38	VFSL731^∗^	2	4y1m	O26	<10	–	+	+	+
		VFSL473^∗^	4	7y2m	O26	<10	–	+	+	–

*^∗^animals positive for STEC more that once during the 1-year sampling program*.

**Table 3:** Farm B: Month of sampling, numbers of animals sampled, animal code, number of lactations, age of animal, *E. coli* serogroup isolated, shedding status, and virulence characteristics.

Month of Sampling	No. Lactating animals sampled	Animal code	No. of lactations	Age of animal	*E. coli* serogroup isolated	Shedding status CFU/swab	Virulence status of recovered isolates			
							*stx*1	*stx*2	*eae*	*hly*A
September, 2013	39	No STEC detected							
December, 2013	37	VFSL868	2	3y6m	O26	100	–	+	+	–
		VFSL858	2	3y6m	O26	<10	–	+	+	–
		VFSL832^∗^	2	3y8m	O26	10,000	+	+	+	–
February, 2014	37	No STEC detected							
April, 2014	37	VFSL578	6	7y8m	O157	<10	–	+	+	–
		VFSL700^∗^	4	5y8m	O157	<10	–	+	–	–
		VFSL700^∗^	4	5y8m	O26	10,000	+	+	+	+
		VFSL777	3	4y8m	O157	<10	–	+	+	–
		VFSL783	3	4y7m	O157	<10	–	+	+	–
		VFSL770	3	4y8m	O157	<10	–	–	+	+
May, 2014	37	VFSL724	4	5y7m	O157	<10	–	+	+	–
		VFSL780	3	4y7m	O26	100	+	+	+	–
		VFSL832^∗^	2	4y1m	O26	<10	+	+	+	–
		VFSL840	2	3y7m	O26	<10	–	+	+	–
June, 2014	37	VFSL763	3	4y8m	O26	<10	+	+	+	–
		VFSL788	3	4y7m	O26	<10	–	+	+	–
		VFSL646	5	6y8m	O157	10	–	+	+	–
		VFSL633	5	6y8m	O157	10,000	–	+	–	–

*^∗^animals positive for STEC more than once during the 1-year sampling program.*

Farm A: 15 of the 305 (5%) recto-anal swabs were *E. coli* O157 positive, 13 of those were confirmed as STEC as they encoded *stx1* and/or *stx2* (**Table 2**). All 13 isolates were positive for the adhering and effacement (*eae*) gene. Two of the isolates were *stx* negative but positive for *eae* (VFSL473 & 434). Five STEC O26 were recovered, all were *stx2* and *eae* positive. Two STEC O26 strains that were additionally positive for haemolysin gene (*hlyA*) were recovered from the same animal (VFSL731) (**Table 2**). One super-shedder (VFSL537) was identified shedding STEC O26 at 10,000 CFU/swab (**Table 2**). In addition, one animal was found to be shedding STEC O157 over three consecutive months (VFSL887).

One animal was colonized with *E. coli* O157 in January, 2014 and STEC O26 in July, 2014 (VFSL473).

One animal was intermittently shedding STEC O157 in November, 2013 and again in May, 2014 (VFSL531) and one STEC O26 in November, 2013 and July, 2014 (VFSL731) (**Table 2**). Additional data on the animals being sampled showed that the age range of positive animals was 2 years 6 months to 12 years 7 months (three were first time calvers) body condition between 3 and 4. A 12-year-old animal (VFSL165) was on her 11th lactation, with the remaining animals between 1 and 6 lactations; the number of days in milk is recorded as between 126 and 291. The super-shedder identified in the herd (VFSL537) was 3 years 3 months old on her second lactation, with a body score of 3 and was 184 days in milk, a SCC of 44,000 and a percentage protein of 3.53 (**Table 2**).

Farm B: eight of the 224 (3.5%) recto-anal swabs were *E. coli* O157 positive, seven being denoted STEC. Five strains were *stx2* and *eae* positive (VFSL578; 777; 783; 724; 646). The *stx* negative strain was *eae* and *hlyA* positive (VFSL770) and two strains were *stx2* (only; VFSL633 & 700) (**Table 3**). Nine STEC O26 were recovered, all nine were *stx2* and *eae* positive with five of these additionally *stx1* positive. One strain encoded all four virulence factors (VFSL700) interestingly this was one of the super-shedders. Three super-shedders were identified (VFSL832; 700 and 633) two shedding STEC O26 and one shedding STEC O157 at 10,000 CFU/swab (**Table 3**). In addition, one of the super-shedders was intermittently shedding STEC O26 in December, 2013 but was shedding very low numbers of STEC O26 in May, 2014 (VFSL832). One animal colonized both STEC O157 and STEC O26 and found to be super-shedding the STEC O26 (VFSL700). The age range of the animals on Farm B was 3 years 6 months to 7 years 8 months, no first time calvers; lactation’s ranged from 2 to 6. Days in milk ranged from 132 to 229. The ages of the three super-shedders was 3 years 8 months (two lactations); 5 years 8 months (four lactations) and 6 years 8 months (five lactations) all had body condition scores of 3 (**Table 3**).

No STEC was recovered from any of the water, milk filter, or raw milk samples from either farm.

## Discussion

To our knowledge this is the first study investigating active super-shedding in dairy cattle in Ireland and the first internationally to examine for super-shedding of O26. This study addressed the carriage and shedding of STEC O157 and O26 in two dairy herds and particularly their level of excretion, i.e., low (<10 – 100 CFU/swab); medium (100 – 1000 CFU/swab), or super-shedding (≥10,000 CFU/swab). The results of this study showed low numbers of super-shedders among the animals screened. The majority of the positive animals were low-shedders; two were medium-shedders with evidence of persistent shedding in some animals tested. There was no STEC O157 or O26 recovered from any of the raw milk, water, or milk filter samples analyzed.

The study identified four super-shedders over the 12-months, in December, 2013; January, 2014; April, 2014 and June, 2014 (**Tables 2** and **3**). Only a single super-shedder was identified in Farm A in January, 2014. The other three animals from Farm B were found to be super-shedding in December, 2013; April, 2014 and June, 2014. Each animal was identified as super-shedding only once during the study. For all animals regardless of shedding status, no seasonality could be determined as recovery of these pathogens occurred over every month of the year (**Tables 2** and **3**). No STEC was recovered from any of the water, milk filter or raw milk samples during the study.

The frequency of super-shedding in a herd is not well understood; a study on super-shedding in 60 heifers in the US reported 3.8% prevalence (Cobbold et al., 2007). A Canadian study on feed-lot cattle recorded 25% (Cernicchiaro et al., 2010) and a German study on dairy herds found 10% were super-shedders (Menrath et al., 2010). Our study identified four super-shedders from 529 animals tested (0.8%). To further our understanding on these results our collaborators in the project (Teagasc) will examine the entire micro-flora of the RAJ from the STEC super-shedding animals identified along with the animals shedding STEC (but not super-shedders) and a control group (non shedders) using a novel 16 s gene-based compositional metagenomic approach to assess the composition and proportion of microbes present at that time. These data should provide knowledge and a broader understanding of this phenomenon.

The presence of these pathogens in a herd even at low numbers may give rise to increased animal to animal and animal to environment transmission. In this present study, the two different serogroups examined colonized one single animal at the same time, and this has been previously reported in a study that showed, three different serogroups colonized one animal (Blanco et al., 1996). Interestingly, the animal which was colonized by both STEC O157 and O26 was super-shedding STEC O26. To our knowledge this is the first report of STEC O26 super-shedding in cattle, thus it is interesting to note that *E. coli* O26 is now the most common STEC serogroup in human illness in Ireland (Health Protection Service Centre [HPSC], 2014).

Virulence screening of the recovered isolates showed that all isolates contained, between one and four of the virulence genes that are commonly associated with human disease (Madic et al., 2011; Bondari et al., 2015). It is essential to monitor ruminants to evaluate the risk associated with STEC infections in humans. Although, this study did not recover any STEC from the raw milk samples, other studies have recovered these pathogens from raw milk and raw milk products (Allerberger et al., 2003; Rangel et al., 2005; Schrijver et al., 2008; Madic et al., 2011) these raw products are associated with higher risk of STEC infection due to the survival of the organism during the manufacturing process. Elhadidy and Álvarez-Ordóñez (2016) applied a total of seven different stresses including, starvation, freeze-thaw, acid, heat, cold, osmotic and oxidative, to two different genotypes of *E. coli* O157:H7, the authors found multi stress resistance in the strains most frequently associated with human disease cases.

The results from the questionnaire showed that neither farm families consumed raw milk; one had a private water supply (Farm A) and one a public water supply (Farm B). Animals on both farms were housed between November and March each year and fed a diet of concentrates during the housed period. Increased awareness of the potential public health implications of this pathogen and the methods for its control, particularly at farm level, were discussed with each farmer (and some farm family members) and the PVPs during the visits. In the event of a positive result, advice was given verbally on personal hygiene and best practice on the farm to prevent the spread of STEC. As outlined in Food Research Ireland: Department of Agriculture (2011) the Government strategy links the sustainability of Ireland’s food sector to its food safety performance and thereby protecting the consumer from serious pathogens (Food Harvest, in press).

This study, although confined to two herds links directly with current strategies by providing knowledge on the risk posed by cattle shedding STEC in large numbers. Eliminating high-level fecal excretion of STEC at farm level may reduce the prevalence of the organism in the host and in-turn reduces the risk of human infection. The study used a robust method to identify super-shedders, once detected a possible strategy may be to remove the super-shedder from the herd prior to movement of animals or slaughter, thus, protecting public health. In addition, measures should be considered for slurry treatment prior to spreading, in tandem with a review of intensive grazing systems, stocking densities and management of the grazing platforms.

Internationally recognized experts strongly advocate a multi-hurdle approach toward minimizing the risk presented by STEC O157 and other Shiga-toxin producing organisms (Collins and Wall, 2004; Matthews et al., 2006b). A further broader study on super-shedding of significantly more farms with a larger number of animals has now been completed and a publication is in preparation (Murphy et al., manuscript in preparation). This study highlights the need for further surveillance to assess the potential for environmental contamination and food chain security.

## Ethical Statement

Standard practices of animal care and use were applied to animals sampled in this project. Research protocols were approved by the Veterinary Department, Cork. County Council, Cork, Ireland.

## Author Contributions

GD, MM, and JB conceived and designed the study. BM, JB, and DC selected the herds and collected samples. BM and EM carried out the laboratory work. BM, EM, and SF analyzed and interpreted the data. BM and MM wrote the manuscript. All authors critically revised and approved the final manuscript

## Conflict of Interest Statement

The authors declare that the research was conducted in the absence of any commercial or financial relationships that could be construed as a potential conflict of interest.

## Footnotes

1 http://www.ncbi.nlm.nih.gov/BLAST

2 http://www.genscript.com

3 http://www.eurofinsgenomics.com
